# Misdiagnosis, detection rate, and associated factors of severe psychiatric disorders in specialized psychiatry centers in Ethiopia

**DOI:** 10.1186/s12991-021-00333-7

**Published:** 2021-02-02

**Authors:** Getinet Ayano, Sileshi Demelash, Zegeye yohannes, Kibrom Haile, Mikiyas Tulu, Dawit Assefa, Abel Tesfaye, Kelemua Haile, Melat Solomon, Asrat Chaka, Light Tsegay

**Affiliations:** 1 Research and Training Department, Amanuel Mental Specialized Hospital, Addis Ababa, Ethiopia; 2 School of Public Health, Curtin University, Perth, WA Australia; 3 Ethiopian Public Health Institute, Addis Ababa, Ethiopia; 4 Department of Medicine, Hawassa University, Hawassa, Ethiopia; 5 Department of Psychiatry, Axum University, Axum, Ethiopia

**Keywords:** Severe psychiatric disorder, Misdiagnosis, Schizoaffective disorder, Bipolar disorder, Depressive disorder, Schizophrenia

## Abstract

**Background:**

There are limited studies on the prevalence of misdiagnosis as well as detection rates of severe psychiatric disorders in specialized and non-specialized healthcare settings. To the best of our knowledge, this is the first study to determine the prevalence of misdiagnosis and detection rates of severe psychiatric disorders including schizophrenia, schizoaffective, bipolar, and depressive disorders in a specialized psychiatric setting.

**Method:**

In this cross-sectional study, a random sample of 309 patients with severe psychiatric disorders was selected by systematic sampling technique. Severe psychiatric disorders were assessed using the Structured Clinical Interview for DSM-IV (SCID). The potential determinates of misdiagnosis were explored using univariable and multivariable logistic regression models, adjusting for the potential confounding factors.

**Result:**

This study revealed that more than a third of patients with severe psychiatric disorders were misdiagnosed (39.16%). The commonly misdiagnosed disorder was found to be a schizoaffective disorder (75%) followed by major depressive disorder (54.72%), schizophrenia (23.71%), and bipolar disorder (17.78%). Among the patients detected with the interview by SCID criteria, the highest level of the correct diagnosis was recorded in the medical record for schizophrenia (76.29%) followed by bipolar (72.22%), depressive (42.40%), and schizoaffective (25%) disorders with detection rate (sensitivity) of 0.76 (95% CI 0.69–0.84), 0.42 (95% CI 0.32–0.53), 0.72 (95% CI 0.60–0.84), and 0.25 (95% CI 0.09–0.41), respectively for schizophrenia, depressive, bipolar, and schizoaffective disorders. Patients with bipolar disorder were more likely to be misdiagnosed as having schizophrenia (60%), whereas schizophrenic patients were more likely to be misdiagnosed as having bipolar disorder (56.25%) and patients with depressive disorders were more likely to be misdiagnosed as having schizophrenia (54.72%). Having a diagnosis of schizoaffective and depressive disorders, as well as suicidal ideation, was found to be significant predictors of misdiagnosis.

**Conclusion:**

This study showed that roughly four out of ten patients with severe psychiatric disorders had been misdiagnosed in a specialized psychiatric setting in Ethiopia. The highest rate of misdiagnosis was observed for schizoaffective disorder (3 out of 4), followed by major depressive disorder (1 out of 2), schizophrenia (1 out of 4), and bipolar disorders (1 in 5). The detection rates were highest for schizophrenia, followed by bipolar, depressive, and schizoaffective disorders. Having a diagnosis of schizoaffective and depressive disorders as well as suicidal ideation was found to be significant predictors of misdiagnosis.

## Background

Epidemiological evidence suggests severe mental disorders such as schizophrenia, schizoaffective, bipolar, and depressive disorders are the major contributors to the global burden of disease [1–5]. Schizophrenia is considered the most severe and heritable disorder affecting 0.4% of the general population [6–8]. Major depressive disorders are the most common and the largest contributor to the global burden of disease with a prevalence of 4.7% study [9, 10]. Bipolar disorder is the other common severe psychiatric disorder with a mean prevalence estimate of 1% in the general population [11].

A substantial body of evidence shows that there are no pathognomonic signs and symptoms in psychiatric disorders [12–14]. The most common and defining symptoms in one disorder could occur in the other distinct category of mental disorders [13]. For example, the commonest symptoms of schizophrenia such as hallucination and delusion may occur in the other categories of severe psychiatric disorders such as bipolar and depressive disorders. In support of this view, studies also reported that a significant proportion of patients with depressive and bipolar disorders had psychosis symptoms [14–16]. Additionally, as many as 60% of schizophrenic patients had comorbid depression in addition to the main schizophrenic symptoms [17–19].

Moreover, research over the past several years has reported that many of the severe psychiatric disorders mimic symptoms and diagnostic criteria of each other, and despite the valuable standard and validated diagnostic criteria [20, 21], accurate diagnosis of those severe psychiatric disorders can be challenging [22]. Thus, many patients with severe psychiatric disorders were commonly misdiagnosed in primary as well as specialized health care settings [23, 24]. For example, epidemiologic studies show that as many as 76.8% of patients with bipolar disorders [24] and 50% of patients with depressive disorders were misdiagnosed [24]. Depression was found to be the most likely misdiagnosed mental disorder instead of bipolar disorder and bipolar disorder was most likely misdiagnosed with depressive disorders [24, 25].

Several factors contributed to a considerably high magnitude of misdiagnosis of severe psychiatric disorders. Firstly, the fact that the diagnosis of psychiatric disorders heavily depend on history taking [13, 26]. Secondly, there exists a significant overlap of symptoms across the disorders [12–14, 27]. Thirdly, instability of the psychiatric symptoms across the disorders including delusion, hallucination, and other symptoms [28, 29]. Fourthly, the experience and knowledge of the professionals involved. Fifthly, the severity and complexity of the presentation [30, 31]. Sixthly, not routinely going through the diagnostic criteria during the process of an initial diagnosis [32].

However, worldwide, there are a few studies on the prevalence of misdiagnosis as well as detection rates of severe psychiatric disorders in specialized and non-specialized healthcare settings. To the best of our knowledge, this is the first study to determine the prevalence of misdiagnosis and detection rates of severe psychiatric disorders including schizophrenia, schizoaffective, bipolar, and depressive disorders in a specialized psychiatric setting.

## Methods

### Participants and study design

In this cross-sectional study, a random sample of 320 patients with severe psychiatric disorders from the outpatient clinic of Amanuel Mental Specialized Hospital was invited to participate. This hospital is the only psychiatric hospital in Ethiopia. Data were collected between May and July 2017. The participant had to meet the following criteria: (1) adults (age over 18 years); (2) positive for severe psychiatric disorders by the structured clinical interview for DSM-ΙV-TR axis Ι disorders (SCID) criteria; (3) having the capacity to consent.

### Sampling procedure

This study is part of a comorbidity study conducted in a clinical setting in Ethiopia. For this study, the sample size was estimated using Epi-info version 7 with a 95% CI 5% margin of error, and taking the prevalence of comorbid physical conditions in people with severe psychiatric disorders nearly 80% [33, 34] and assuming normally distributed data, we employed the following formulae calculate the sample size for a single proportion at $\propto =$ 0.05:

$$n = \frac{{\left( {Z\frac{\alpha }{2}} \right)^{2} p\left( {1 - p} \right)}}{{d^{2 } }},$$

where *n* = sample size; *Z* $\frac{\propto }{2}$= significance level at $\propto =$ 0.05; *p* = expected proportion of comorbidity among patients with severe mental illness = 80%; *d* = margin of error = 0.05. Therefore,

$$n = \frac{{\left( {1.96} \right)^{2} 0.5\left( {1 - 0.5} \right)}}{{0.05^{2 } }}\; = \;246.$$

Considering a 30% non-response rate a total sample of 320 patients with severe psychiatric disorders were included [35].

We used a systematic random sampling technique to reach each study participant. The sampling interval was identified by dividing the total patients with severe psychiatric disorders who had the treatment and follow-up during the data collection period by the total sample size which was 11. To select the first participant, we utilized the lottery method and the remaining participants were selected at a regular interval as suggested by a systematic sampling method [36].

### Measures

In the present study, the sociodemographic and clinical characteristics such as age, sex, residence, marital status, educational status, ethnicity, religion, suicide, duration of the illness, history of relapse, and hospitalizations were collected from each of the participants. The study participants were then interviewed using the structured clinical interview for DSM-ΙV-TR axis Ι disorders (SCID) [37] for DSM = IV severe psychiatric disorders criteria. SCID is a diagnostic instrument used to assess DSM-IV-axis I disorders (major mental disorders) and it is extensively used in Ethiopia to assess psychiatric disorders in previous studies [38, 39]. All data was collected using trained assessors (masters level psychiatry professionals) assigned to evaluate the participants based on SCID criteria.

### Data quality control

In the current study, to assure the quality of the data, the data collector (psychiatry professionals) who have adequate knowledge and experience about DSM-IV-TR were recruited. Additionally, training was delivered on how to complete the questionnaire, sampling procedure, inclusion criteria, ethical consideration, data collection procedure as well as the details of SCID for the data collectors and supervisors. The questionnaire was pretested before the actual data collection and the necessary modification was made. Two supervisors followed the data collectors and the necessary correction was undertaken when needed. The collected data were reviewed and checked for completeness and relevance by the supervisor and principal investigator each day.

### Statistical analysis

All statistical analysis has been carried out using Stata (version 16). Categorical variables were summarized using counting (frequency) and expressed as a percentage. Continuous variables were expressed as mean (standards deviations). Regarding our outcome of interest, to calculate the rates of misdiagnosis the previous diagnosis has been taken from the chart (professional diagnosis) and the current diagnosis was based on SCID criteria. The results were then expressed as frequency and percentage. A paired Chi-square test has been employed to assess the detection rates and the results were presented as a percentage with the respective P-value. All the reported probabilities were two-sided.

Concerning the associated factors, bivariate and multivariate logistic regression was conducted to look at the association between outcome and explanatory variables. The strength of the association was measured by OR with 95% CI and *p*-value less than 0.05 was considered as statistically significant.

### Ethical consideration

The human research and ethics committee (HREC) of Amanuel Mental Specialized Hospital (Research and training department) reviewed and approved the study in accordance with the given roles and national research ethics guidelines [40]. The study participants provided written informed consent after a clear and detailed explanation of the purpose, objectives, significance, benefits, and harms of participation, as well as confidentiality of the collected information to each of the study participants. During the period of the data collection, the investigator, supervisor, and data collectors followed the code of ethics and obeyed the rules and regulations of the hospital. Privacy was kept confidential at the time of data collection.

## Results

### Sociodemographic characteristics of the participants

The sociodemographic characteristics of the participant are summarized in the table in Table 1. A total of 309 participants with severe psychiatric disorders including schizophrenia (*n* = 135), schizoaffective (*n* = 28), bipolar (*n* = 54), and depressive (*n* = 92) disorders were involved yielding a response rate 96.56%. The mean (SD) age and duration of the participants were 36.19 (10.45) and 10.04 (8.66) years, respectively. Nearly two-thirds of the participants 202 (65.37%) were males, 202 (65.37%) were single, 118 (38.19%) attended secondary school, nearly half 157 (51.47%) were Orthodox Christians, and the majority of the participants 235 (76.05%) were from urban areas.

**Table 1:** Sociodemographic characteristics of the participants with severe psychiatric disorders in Addis Ababa, Ethiopia (*n* = 309)

Characteristics	Frequency	Percentage
Sex		
Male	202	65.37
Female	107	34.63
Age		
30 or less	110	35.6
30 to 40	106	34.3
41 and more	93	30.1
SPD type		
Schizophrenia	135	43.69
Bipolar disorder	54	17.48
MDD	92	29.77
Schizoaffective disorders	28	9.06
Educational status		
Uneducated	30	9.71
Primary	103	33.33
Secondary	118	38.19
Higher	58	18.77
Ethnicity		
Amhara	95	30.74
Gurage	82	26.54
Oromo	91	29.45
Others	41	13.27
Religion		
Muslim	87	28.16
Orthodox	157	51.46
Protestant	57	18.54
Others	6	1.94
Marital status		
Single	202	65.37
Married	74	23.95
Divorcee/widowed	33	10.68
Residence		
Urban	235	76.05
Rural	74	29.95

### The prevalence of misdiagnosis in people with severe mental disorders

In this study, roughly four out of ten patients with severe psychiatric disorders were misdiagnosed (prevalence = 39.16%; 95% CI 33.70–44.60). Concerning the specific severe mental disorders, more than half of people with MDD were misdiagnosed (57.60%). On the other hand, less than 50% of the patients with MDD identified by the SCID criteria (*n* = 92) were correctly identified by the professionals (42.40%). The differences between the SCID and professional chart diagnoses were statistically significant (*p*-value < 0.0001) (Table 2 and Additional file 1). Major depressive disorder was most likely diagnosed as schizophrenia (*n* = 29, 54.72%) followed by bipolar (*n* = 21, 39.62%) and schizoaffective (*n* = 3, 5.66%) disorders.

**Table 2:** Misdiagnosis of patients with severe mental disorders in central Ethiopia, *n* = 309

Disorder (SPD)	SCID diagnosis	Correct chart diagnosis	Misdiagnosis	Remarks	Paired test (2 tailed), *p* value
	*F*	*F* (*p*)	*F* (*p*)		
MDD	92	39 (42.39)	53 (57.60)	Underdiagnosis	< 0.0001
SCZ	135	103 (76.30)	32 (23.70)	Underdiagnosis	< 0.0001
BD	54	39 (72.22)	15 (27.78)	Underdiagnosis	0.0001
SCZAF	28	7 (25)	21 (75)	Underdiagnosis	< 0.0001

*SPD* severe psychiatric disorder, *MDD* major depressive disorder, *SCZ* schizophrenia, *BD* bipolar disorder, *SCAZAF* schizoaffective disorder, *F* frequency, *P* percentage

We also found that almost a quarter of schizophrenic patients were misdiagnosed by professionals (23.71%) with a significant difference between the SCID and professional chart diagnosis (*p*-value < 0.0001) (Table 2 and Additional file 1). Schizophrenia was most likely diagnosed as bipolar disorder (56.25%) followed by major depressive ((*n* = 13, 40.63%) and schizoaffective ((*n* = 1, 3.12%) disorders.

Similarly, this study revealed that a notable proportion of patients with bipolar disorder have received a misdiagnosis (17.78%). The variation between the SCID and chart diagnosis is statistically significant (*p*-value < 0.0001) (Table 2 and Additional file 1). Bipolar disorder was most likely diagnosed as schizophrenia ((*n* = 9, 60%) followed by major depressive ((*n* = 15, 30%) and schizoaffective ((*n* = 1, 10%) disorders.

With regard to schizoaffective disorders, the current study revealed a considerably higher magnitude of misdiagnosed schizoaffective disorder (75%) with a significant difference between the SCID and professional chart diagnosis (*p*-value < 0.0001) (Table 2 and Additional file 1). Schizoaffective disorder was most likely diagnosed as schizophrenia (*n* = 15, 71.72%) followed by bipolar (*n* = 15, 23.80%) and major depressive disorder ((*n* = 1, 4.76%) disorders.

### The detection rate of people with PSD by professionals (correct chart diagnosis)

In the current study, among the patients detected with the interview by SCID criteria, the highest level of the correct diagnosis was recorded in the medical record for schizophrenia (*n* = 103, 76.29%) followed by bipolar ((*n* = 39, 72.22%), depressive ((*n* = 39, 42.40), and schizoaffective ((*n* = 7, 25%) disorders with detection rate (sensitivity) of 0.76 (95% CI 0.69–0.84), 0.42 (95% CI 0.32–0.53), 0.72 (95% CI 0.60–0.84), and 0.25 (95% CI 0.09–0.41), respectively, for schizophrenia, depressive, bipolar, and schizoaffective disorders.

### Factors associated with misdiagnosis of severe psychiatric disorders

The present study revealed that having suicidal ideation, schizoaffective and major depressive disorders were positively and significantly associated with misdiagnosis in people with severe psychiatric disorders. The odds of having misdiagnosis was increased by 4.22 for MDD [AOD = 4.22 (95% CI 1.69–10.56)]. Similarly, the odds of misdiagnosis were increased by 12.39 for schizoaffective disorder [AOR = 12.39 (95% CI 4.50–34.16)] and by 2.19 for suicidal ideation [AOR = 2.19 (95% CI 1 1.24–3.87)] (Table 3).

**Table 3:** Factors associated with suicide in people with severe mental disorders, Addis Ababa, Ethiopia

Characteristics	Misdiagnosis		Crude odds ratio (95% CI)	Adjusted odds ratio (95% CI)
	Yes	No		
Gender				
Male	83	119	1.27 (0.80–2.06)	1.52 (0.85–2.71)
Female	38	69	1	1
Age				
18–30	46	64	1.09 (0.62–1.91)	1.17 (0.58–2.36)
31–40	38	68	0.86 (0.48–1.50)	1.01 (0.52–1.96)
≥ 40	37	56	1	1
Educational status				
No formal education	15	15	1.63 (0.67–3.99)	1.46 (0.49–4.34)
Primary	38	65	0.96 (0.49–186)	0.83 (0.38–1.78)
Secondary	46	72	1.05 (0.55–1.96)	1.13 (0.54–2.34)
Higher	22	36	1	1
Residence				
Rural	34	40	1	1
Urban	87	148	1.45 (0.85–2.45)	1.49 (0.78–2.82)
Marital status				
Single	79	123	1	1
Married	30	44	1.07 (0.62–1.83)	0.87 (0.45–1.66)
Divorce/widowed	12	21	0.89 (0.41–1.96)	1.15 (0.48–2.82)
Type of SPD				
MDD	53	39	4.37 (2.47–7.76)	4.22 (1.69–10.56)*
BPD	15	39	1.24 (0.61–2.53)	1.24 (0.57–2.68)
SCZAF	21	7	9.66 (3.76–24.79)	12.39 (4.50–34.16)**
SCZ	32	103	1	1
MDD psychosis				
Yes	38	26	2.85 (1.62–5.02)	1.14 (0.44–2.95)
No	83	162	1	1
Suicidal ideation				
Suicide	87	104	2.07 (1.27–3.37)	2.19 (1.24–3.87)*
No suicide	34	84	1	1
Relapse				
Relapsed	90	169	1.11 (0.66–1.86)	1.41 (0.75–2.62)
No relapse	31	52	1	
Admission				
Admission	78	118	1.07 (0.69–1.73)	1.23 (0.70–2.16)
No admission	43	70	1	1

*SCZ* schizophrenia, *MDD* major depressive disorder, *BD* bipolar disorder, *SCZAF* schizoaffective disorder

^*^Significant association (*p*-value < 0.05); **significant association (*p*-value < 0.001)

## Discussion

### Main findings

To the best of our knowledge, this is the first study to determine the prevalence of misdiagnosis and detection rates of severe psychiatric disorders including schizophrenia, schizoaffective, bipolar, and depressive disorders in a specialized psychiatric setting. The results of our assessment revealed that a notably high proportion of people with severe psychiatric disorders were misdiagnosed and the detection rates of the distinct categories of severe psychiatric disorders were relatively low. Regarding specific disorders, roughly three out of four and one out of two patients with schizoaffective and major depressive disorders, respectively, were misdiagnosed. We also found that roughly one in four and one in five of patients with schizophrenia and bipolar disorder, respectively, were misdiagnosed. In addition, the current study demonstrated remarkably low detection rates of schizoaffective disorder (25%), depressive disorder (42.40%), bipolar disorder (72.22%) as well as schizophrenia (76.29%). Having a diagnosis of schizoaffective and depressive disorders as well as suicidal ideation was found to be significant predictors of misdiagnosis. This study adds to scant knowledge that a significant proportion of patients with severe mental disorders have been misdiagnosed and undetected even in a specialized mental health setting. Considerably higher misdiagnoses and lower detection rates indicate the problem is a burning public health issue that warrants urgent attention by the government, the institutions (hospitals), as well as the professionals assessing the patients. The findings also suggest that strong effort is needed to reduce the rates of misdiagnoses as well as increase the detection rates of the disorders, which are associated with a wide range of negative outcomes, including severe disability and suffering from the symptoms [32].

### The possible reasons for the misdiagnosis

There are a wide range of explanations for the observed considerable level of misdiagnosis of severe psychiatric disorders. First, failure to appreciate the significance of extensive and expert psychiatric history taking by the professionals. In support of this view, evidences indicated that psychiatric history taking is the most important component in the evaluation and care of patients with mental disorder [41, 42]. Additionally, psychiatric history taking is considered as a part of the treatment process (first stage of treatment process) where we collect important information for final psychiatric diagnosis [13]. Therefore, it is strongly recommended that adequate time must be given in taking a history from the patients with an average length time roughly up to 45 min, but the length of time varies depending on the setting, the complexity of the presentation, the purpose of the interview and other factors (including additional assessment tools for the quality or other purposes of services) [13, 41, 42]. However, according to the unpublished study report that assessed the length of time for psychiatric assessment in the same setting in Ethiopia, the average length of time for psychiatric evaluation was only five minutes. Secondly, the severity and complexity of the presentation might be the other reasons for the misdiagnosis. This is because as the study was conducted in a tertiary hospital, the patients were more likely to serve and referred from the different areas of the country, and the more severe the disorder the more likely to be the overlapping presentation leading to misdiagnosis [43, 44]. In support of the above explanation, the current study indicated that a significantly higher proportion of people with bipolar (88.89%) and depressive (69.56%) disorders had overlapping psychotic symptoms and nearly one in five of patients with schizophrenia had depressive symptoms during evaluation. Thirdly, the higher rates of misdiagnosis could be attributed to the low level of clinical experience, knowledge about the psychiatric disorders and diagnostic criteria’s, as well as the subjective nature of the diagnosis due to the absence of any supporting laboratory evidence in the diagnosis of psychiatric disorders [8, 45–47]. This is because in Ethiopia professionals without psychiatry specialty as well as diploma and degree level trained psychiatry nurses were involved in the care, diagnosis, and treatment of patients with mental disorders because of the scarcity of specialized manpower [48]. The perception of the professionals that inaccurate diagnoses might benefit the patients or it has been assigned due to diagnostic considerations (for example, the presence of negative symptoms for schizophrenia) are the other possible reasons for the increased rates of misdiagnosis [49]. Finally, the diagnostic instability and the change from one disorder to the other disorder over time might be the other possible attributing factor for the misdiagnosis. Because in the current study the average duration of the disorder was 10 years and epidemiologic evidence indicates that as many as 50% of patients with bipolar disorder had a shift to non-bipolar disorder at least once over 10 years [50].

### Comparing with the existing literature

In the present study, the commonly misdiagnosed disorder was found to be a schizoaffective disorder (75%) followed by major depressive disorder (54.72%), schizophrenia (23.71%), and bipolar disorder (17.78%). The vast majority of patients with schizoaffective disorders were misdiagnosed as having schizophrenia (53.57%). The remaining patients were misdiagnosed as having bipolar (17.86%) and depressive disorders (3.57%). The possible reason for higher rates of misdiagnoses of schizoaffective disorder could be the clinical presentation and the required criteria to diagnose schizoaffective disorders are more complex containing similar symptoms and diagnostic criteria to both schizophrenia and mood disorder episodes (manic or depressive episodes) [51]. Additionally, the criteria to diagnose schizoaffective disorder are more strict requiring the presence of psychotic symptoms occurring for at least 2 weeks without prominent mood episodes [45]. Our findings are supported by a previous epidemiology study that found the least interrater reliability and low diagnostic congruence for schizoaffective disorders as compared with schizophrenia, bipolar, and major depressive disorders [52].

The findings of the current study indicating bipolar disorder as the least misdiagnosed disorders as compared to the other severe psychiatric disorders are supported by the validation study that identified bipolar disorder as the disorder with the highest degree in both diagnostic congruence and interrater reliability as compared with schizophrenia, schizoaffective, and depressive disorders [52]. However, the rate of bipolar disorder misdiagnosis was remarkably lower than the results of the previous studies conducted in China (76.8%) [24]. The possible reasons for this difference might be due to the variations in the episodes, presenting symptoms as well as the difference in the characteristics the professionals used to evaluate the disorders.

The current study also demonstrated that patients with bipolar disorder were more likely to be misdiagnosed as having schizophrenia (60%), whereas schizophrenic patients were more likely to be misdiagnosed as having bipolar disorder (56.25%). The possible reasons might be due to the severity of the bipolar disorder in the current study where 88.89% of bipolar disorders had psychotic features, which is essentially the core and defining symptoms of schizophrenia [53]. Supporting this view a study found that the presence of psychotic symptoms in bipolar patients was associated with increased rates of misdiagnosis [54] and the majority of patients with bipolar with psychotic features were misdiagnosed as having psychotic disorders including schizophrenia [55, 56]. These findings are in contrast to the previous scientific evidence that reported depression (70.6%) as the most likely misdiagnosed disorder instead of bipolar disorders [24]. The other possible reason for the difference is that in the present study nearly half of the participants with bipolar disorder had only manic episodes in their lifetime which had the presenting symptoms more resembling the symptoms of schizophrenia than the symptoms of depressive disorders.

We also found that patients with depressive disorder were more likely to be diagnosed as having schizophrenia (54.72%). The possible reasons for this might be the presence of psychotic symptoms (66.67%) in addition to the depressive symptoms in patients with major depressive disorders in the current study.

Moreover, having a diagnosis of schizoaffective [AOR = 12.39 (95% CI 4.50–34.16)], and depressive disorders [AOD = 4.22 (95% CI 1.69–10.56)], as well as suicidal ideation [AOR = 2.19 (95% CI1 1.24–3.87)], was found to be significant predictors of misdiagnosis. These findings were in agreement with similar previous studies [24, 30]. One of the possible reasons for an increased risk of misdiagnosis among patients with suicidal ideation could be due to the presence of extensive evidence that shows depressive disorders as disorders with higher rates of suicide than other psychiatric disorders [57–59], which potentially make professionals draw more attention to the disorders with high rates of suicide (depressive disorders or disorders with a depressive episode) during a routine psychiatric assessment. The other possible reason for the increased risk of misdiagnosis in patients having suicidal ideation may be the severity of the disorders. Supporting this view studies suggest that illness severity and the presence of comorbidity were the major factors contributing to increased risk of suicide among patients with severe mental illness [60, 61].

Consistent with previous epidemiologic studies [24], this study revealed that the detection rates were highest for schizophrenia, followed by bipolar, depressive, and schizoaffective disorders.

### Implications for future research and clinical practice

The current study had some implications for future research as well as clinical practice. First, this study showed that the misdiagnosis and poor detection rates were remarkably higher in patients with severe psychiatric disorders especially for schizoaffective and depressive disorders, which need future robust studies confirming the observed prevalence and evaluating the possible reasons for the highest prevalence. Second, in the current studies, we included specific categories of mental disorders by distributing the overall sample size calculated for severe psychiatric disorders. This shows that the sample for each disorder may be below the estimated magnitude for distinct categories of the disorders. So future studies addressing this issue are warranted. Thirdly, attention needs to be given to possibly reduce the extensive level of misdiagnoses by the concerned body with the possibilities of implementing continuous medical education (CME) [62, 63], so that the patients will be protected from suffering from persistence symptoms as well as unnecessary and inappropriate drug use consequently leading to an increased level of severity of the disorders due to misdiagnosis and side effects of drugs. Fourth, future studies investigating the potential determining factors for an increased rate of misdiagnoses focusing on the specific disorders and samples from both clinical and community settings are warranted. Fifth, observational, as well as experimental studies assessing the possible factors that increase the detection ability of the professionals, are also needed. Finally, as suggested by the existing scientific evidence routinely going through the diagnostic criteria during the process of an initial diagnosis is advised to reduce the rates of misdiagnoses [64, 65] as well as the negative outcomes of misdiagnoses, including the probability of receiving inadequate or inappropriate diagnoses which in turn are linked with severe disability and suffering from the symptoms [32, 66].

### Strengths and limitations

This study had several strengths: (1) being the first study to estimate and compare the level of misdiagnosis and detection rates across severe psychiatric disorders such as schizophrenia, schizoaffective, bipolar, and depressive disorders; (2) the use of standard and diagnostic instruments (SCID) to examine severe psychiatric disorders; (3) inclusion of the participants from a well-defined catchment area and assessing the indicators of severity such as psychosis in bipolar and depressive disorders which are the possible reasons for a remarkably high magnitude of misdiagnosis.

However, the current study had also some limitations: first, due to the cross-sectional nature of the study factors associated with misdiagnosis may not imply causality. Second, the possibility of recall bias due to the retrospective nature of the study might impact the magnitude of misdiagnosis. Third, our findings were based on participants selected only from clinical settings, which might have been influenced by unexplored area- and community-level factors. Fourth, the inclusion of participants from the clinical setting only limits the generalizability of the results to other participants within a different research environment and socio-economic setting (general population samples).

## Conclusion

In summary, this study revealed that a notably high proportion of patients with severe psychiatric disorders had been misdiagnosed in a specialized psychiatric setting in Ethiopia (four out of ten). The highest rate of misdiagnosis was observed for schizoaffective disorder (75%), followed by depressive disorder (54.72%), schizophrenia (23.71%), and bipolar disorder (17.78%).). The detection rates were highest for schizophrenia, followed by bipolar, depressive, and schizoaffective disorders. Having a diagnosis of schizoaffective and depressive disorders as well as suicidal ideation was found to be significant predictors of misdiagnosis.

Robust longitudinal studies assessing the reasons for the highest level of misdiagnosis as well as identifying the common determinates for the misdiagnosis are warranted. Continuous medical education (CME) and other refreshment trainings are recommended for the professionals.

## Supplementary Information

**Additional file 1.** Rate of misdiagnosis of severe psychiatric disorders at Amanuel Mental Specialized Hospital.

## References

[1] Murray CJ, Lopez AD (1997). Global mortality, disability, and the contribution of risk factors: Global Burden of Disease Study. Lancet.

[2] Murray CJ, Vos T, Lozano R, Naghavi M, Flaxman AD, Michaud C, Ezzati M, Shibuya K, Salomon JA, Abdalla S (2012). Disability-adjusted life years (DALYs) for 291 diseases and injuries in 21 regions, 1990–2010: a systematic analysis for the Global Burden of Disease Study 2010. Lancet.

[3] Ayano G, Tesfaw G, Shumet S (2019). The prevalence of schizophrenia and other psychotic disorders among homeless people: a systematic review and meta-analysis. BMC psychiatry.

[4] Ayano G, Shumet S, Tesfaw G, Tsegay L (2020). A systematic review and meta-analysis of the prevalence of bipolar disorder among homeless people. BMC Public Health.

[5] Murray CJ, Aravkin AY, Zheng P, Abbafati C, Abbas KM, Abbasi-Kangevari M, Abd-Allah F, Abdelalim A, Abdollahi M, Abdollahpour I, Abegaz KH (2020). Global burden of 87 risk factors in 204 countries and territories, 1990–2019: a systematic analysis for the Global Burden of Disease Study 2019. Lancet.

[6] Goldner EM, Hsu L, Waraich P, Somers JM (2002). Prevalence and incidence studies of schizophrenic disorders: a systematic review of the literature. Canadian J Psychiatry.

[7] Saha S, Chant D, Welham J, McGrath J (2005). A systematic review of the prevalence of schizophrenia. PLoS Med.

[8] Ayano G (2016). Schizophrenia: a concise over view on etiology epidemiology diagnosis and management: review on literature. J Schizophrenia Res..

[9] Ferrari AJ, Somerville AJ, Baxter AJ, Norman R, Patten SB, Vos T, Whiteford HA (2012). Global variation in the prevalence and incidence of major depressive disorder: a systematic review of the epidemiological literature. Psychol Med.

[10] Weissman MM, Bland RC, Canino GJ, Faravelli C, Greenwald S, Hwu H-G, Joyce PR, Karam EG, Lee C-K, Lellouch J (1996). Cross-national epidemiology of major depression and bipolar disorder. JAMA.

[11] Clemente AS, Diniz BS, Nicolato R, Kapczinski FP, Soares JC, Firmo JO, Castro-Costa É (2015). Bipolar disorder prevalence: a systematic review and meta-analysis of the literature. Revista Brasileira de Psiquiatria.

[12] Carpenter WT, Strauss JS, Muleh S (1973). Are There pathognomonic symptoms in schizophrenia?: An empiric investigation of Schneider's first-rank symptoms. JAMA Psychiatry.

[13] Sadock BJSV, Ruiz P (2009). Kaplan and Sadock's comprehensive textbook of psychiatry. Lippincott Williams Wilkins.

[14] Lee SH, Ripke S, Neale BM, Faraone SV, Purcell SM, Perlis RH, Mowry BJ, Thapar A, Goddard ME, Witte JS (2013). Genetic relationship between five psychiatric disorders estimated from genome-wide SNPs. Nat Genet.

[15] Dunayevich E, Keck PE (2000). Prevalence and description of psychotic features in bipolar mania. Curr Psychiatry Rep.

[16] Rothschild AJ (2013). Challenges in the treatment of major depressive disorder with psychotic features. Schizophr Bull.

[17] Zisook S, McAdams LA, Kuck J, Harris MJ, Bailey A, Patterson TL, Judd LL, Jeste DV (1999). Depressive symptoms in schizophrenia. Am J Psychiatry.

[18] Dai J, Du X, Yin G, Zhang Y, Xia H, Li X, Cassidy R, Tong Q, Chen D, Teixeira AL (2018). Prevalence, demographic and clinical features of comorbid depressive symptoms in drug naive patients with schizophrenia presenting with first episode psychosis. Schizophr Res.

[19] Gozdzik-Zelazny A, Borecki L, Pokorski M (2011). Depressive symptoms in schizophrenic patients. Eur J Med Res.

[20] Diagnostic Stastistical Manual of Mental Disorder (5 th edition), Fifth edition edn. Wahington, DC: American Psychiatric Association; American Psychiatric Association, 2013.

[21] Organization WH (1992). The ICD-10 classification of mental and behavioural disorders: clinical descriptions and diagnostic guidelines.

[22] Phillips ML, Kupfer DJ (2013). Bipolar disorder diagnosis: challenges and future directions. Lancet.

[23] Wittchen H-U, Mühlig S, Beesdo K (2003). Mental disorders in primary care. Dialog Clin Neurosci.

[24] Shen H, Zhang L, Xu C, Zhu J, Chen M, Fang Y (2018). Analysis of misdiagnosis of bipolar disorder in an outpatient setting. Shanghai Arch Psychiatry.

[25] Hirschfeld RM, Lewis L, Vornik LA (2003). Perceptions and impact of bipolar disorder: how far have we really come? Results of the national depressive and manic-depressive association 2000 survey of individuals with bipolar disorder. J Clin Psychiatry.

[26] Pies R (2007). How "objective" are psychiatric diagnoses?: (guess again). Psychiatry.

[27] Culpepper L (2014). Misdiagnosis of bipolar depression in primary care practices. J Clin Psychiatry.

[28] Winokur G, Scharfetter C, Angst J (1985). Stability of psychotic symptomatology (delusions, hallucinations), affective syndromes, and schizophrenic symptoms (thought disorder, incongruent affect) over episodes in remitting psychoses. Eur Archiv Psychiatry Neurol Sci.

[29] Salvatore P, Baldessarini RJ, Khalsa HM, Amore M, Di Vittorio C, Ferraro G, Maggini C, Tohen M (2013). Predicting diagnostic change among patients diagnosed with first-episode DSM-IV-TR major depressive disorder with psychotic features. J Clin Psychiatry.

[30] Rothschild AJ, Winer J, Flint AJ, Mulsant BH, Whyte EM, Heo M, Fratoni S, Gabriele M, Kasapinovic S, Meyers BS (2008). Missed diagnosis of psychotic depression at 4 academic medical centers. J Clin Psychiatry.

[31] Altamura AC, Goikolea JM (2008). Differential diagnoses and management strategies in patients with schizophrenia and bipolar disorder. Neuropsychiatr Dis Treat.

[32] Nasrallah HA (2015). Consequences of misdiagnosis: inaccurate treatment and poor patient outcomes in bipolar disorder. J Clin Psychiatry.

[33] De Hert M, Correll C, Bobes J, Cetkovich-Bakmas M, Cohen D, Asai I, Detraux J, Gautam S, Möller H-J, Ndetei DM (2011). Physical illness in patients with severe mental disorders. I. Prevalence, impact of medications and disparities in health care. World Psychiatry.

[34] Ayano G (2019). Co-occurring medical and substance use disorders in patients with schizophrenia: a systematic review. Int J Mental Health.

[35] Sesay C, Chien L-Y (2012). Analysis of factors associated with failure to return for an HIV-test result in The Gambia. Afr J AIDS Res.

[36] Suresh K, Thomas SV, Suresh G (2011). Design, data analysis and sampling techniques for clinical research. Ann Indian Acad Neurol.

[37] First M, Spitzer R, Gibbon M, Williams J. Structured clinical interview for DSM-IV axis I disorders, clinician version (SCID-CV) American Psychiatric Press. Washington, DC 1997.

[38] Ayano G, Assefa D, Haile K, Chaka A, Solomon H, Hagos P, Yohannis Z, Haile K, Bekana L, Agidew M (2017). Mental, neurologic, and substance use (MNS) disorders among street homeless people in Ethiopia. Ann Gen Psychiatry.

[39] Duko B, Ayano G, Bekana L, Assefa D (2015). Prevalence and correlates of co-occurring substance use disorder among patients with severe mental disorder at amanuel mental specialized hospital, Addis Ababa Ethiopia. J Neuropsychopharmacol Mental Health.

[40] ESTC: The Ethiopian science and technology commission (ESTC): National research ethics review guideline 2014.

[41] Silverman JJ, Galanter M, Jackson-Triche M, Jacobs DG, Lomax JW, Riba MB, Tong LD, Watkins KE, Fochtmann LJ, Rhoads RS (2015). The American psychiatric association practice guidelines for the psychiatric evaluation of Adults. Am J Psychiatry.

[42] Huntington G (2015). De praestigiis daemonum: the origins of psychiatric history-taking–psychiatry in history. Br J Psychiatry.

[43] Nakamura K, Iga J, Matsumoto N, Ohmori T (2015). Risk of bipolar disorder and psychotic features in patients initially hospitalised with severe depression. Acta Neuropsychiatrica.

[44] Burton CZ, Ryan KA, Kamali M, Marshall DF, Harrington G, McInnis MG, Tso IF (2018). Psychosis in bipolar disorder: does it represent a more "severe" illness?. Bipolar Disord.

[45] American Psychiatric Association (2000). Diagnostic and statistical manual of mental disorders-IV-TR.

[46] Ayano G: Bipolar Disorder: A Concise Overview of Etiology, Epidemiology Diagnosis and Management: Review of Literatures. SOJ Psychol 3 (1): 1–8. *Bipolar Disorder: A Concise Overview of Etiology, Epidemiology Diagnosis and Management: Review of Literatures* 2016.

[47] Ayano G, Assefa D, Haile K, Bekana L (2016). Experiences, strengths and challenges of integration of mental health into primary care in Ethiopia Experiences of East African Country. Fam Med Med Sci Res.

[48] Ayano G (2016). Primary mental health care services in Ethiopia: experiences, opportunities and challenges from East African country. J Neuropsychopharm Ment Health.

[49] Tzur Bitan D, Grossman Giron A, Alon G, Mendlovic S, Bloch Y, Segev A (2018). Attitudes of mental health clinicians toward perceived inaccuracy of a schizophrenia diagnosis in routine clinical practice. BMC Psychiatry.

[50] Ruggero CJ, Carlson GA, Kotov R, Bromet EJ (2010). Ten-year diagnostic consistency of bipolar disorder in a first-admission sample. Bipolar Disord.

[51] Malaspina D, Owen MJ, Heckers S, Tandon R, Bustillo J, Schultz S, Barch DM, Gaebel W, Gur RE, Tsuang M (2013). Schizoaffective disorder in the DSM-5. Schizophr Res.

[52] Cheniaux E, Landeira-Fernandez J, Versiani M (2009). The diagnoses of schizophrenia, schizoaffective disorder, bipolar disorder and unipolar depression: interrater reliability and congruence between DSM-IV and ICD-10. Psychopathology.

[53] Owen MJ, Sawa A, Mortensen PB (2016). Schizophrenia. Lancet.

[54] Altamura AC, Buoli M, Caldiroli A, Caron L, Cumerlato Melter C, Dobrea C, Cigliobianco M, Zanelli Quarantini F (2015). Misdiagnosis, duration of untreated illness (DUI) and outcome in bipolar patients with psychotic symptoms: a naturalistic study. J Affect Disord.

[55] Gonzalez-Pinto A, Gutierrez M, Mosquera F, Ballesteros J, Lopez P, Ezcurra J, Figuerido JL, de Leon J (1998). First episode in bipolar disorder: misdiagnosis and psychotic symptoms. J Affect Disord.

[56] Altamura AC, Goikolea JM (2008). Differential diagnoses and management strategies in patients with schizophrenia and bipolar disorder. Neuropsychiatric Dis Treat.

[57] Duko B, Ayano G (2018). Suicidal ideation and attempts among people with severe mental disorder, Addis Ababa, Ethiopia, comparative cross-sectional study. Ann Gen Psychiatry.

[58] Brådvik L (2018). Suicide Risk and Mental Disorders. Int J Environ Res Public Health.

[59] Ayano G, Tsegay L, Abraha M, Yohannes K (2019). Suicidal ideation and attempt among homeless people: a systematic review and meta-analysis. Psychiatr Q.

[60] Fazel S, Wolf A, Larsson H, Mallett S, Fanshawe TR (2019). The prediction of suicide in severe mental illness: development and validation of a clinical prediction rule (OxMIS). Translat Psychiatry.

[61] Handley T, Rich J, Davies K, Lewin T, Kelly B (2018). The Challenges of predicting suicidal thoughts and behaviours in a sample of rural Australians with depression. Int J Environ Res Public Health.

[62] Mariam M, Bedaso A, Ayano G, Ebrahim J (2016). Knowledge, attitude and factors associated with mental illness among nurses working in public hospitals, Addis Ababa, Ethiopia. J Ment Disord Treat.

[63] Schütze H, Shell A, Brodaty H (2018). Development, implementation and evaluation of Australia’s first national continuing medical education program for the timely diagnosis and management of dementia in general practice. BMC Med Educ.

[64] First MB, Rebello TJ, Keeley JW, Bhargava R, Dai Y, Kulygina M, Matsumoto C, Robles R, Stona A-C, Reed GM (2018). Do mental health professionals use diagnostic classifications the way we think they do? A global survey. World Psychiatry.

[65] Ayano G, Assefa D, Haile K, Chaka A, Haile K, Solomon M, Yohannis K, Adane AA, Jemal K (2017). Mental health training for primary health care workers and implication for success of integration of mental health into primary care: evaluation of effect on knowledge, attitude and practices (KAP). Int J Mental Health Syst.

[66] Ayano G, Duko B (2017). Relapse and hospitalization in patients with schizophrenia and bipolar disorder at the St Amanuel Mental Specialized Hospital, Addis Ababa, Ethiopia: a comparative quantitative cross-sectional study. Neuropsychiatr Dis Treat.

