# Supplementary material for: Misdiagnosis, detection rate, and associated factors of severe psychiatric disorders in specialized psychiatry centers in Ethiopia
Source: Ann Gen Psychiatry. 2021 Feb 2;20:10. doi: 10.1186/s12991-021-00333-7 (PMC7856725; doi:10.1186/s12991-021-00333-7)
Supplement: Supplementary file 1 — Additional file 1. Rate of misdiagnosis of severe psychiatric disorders at Amanuel Mental Specialized Hospital. [file 12991_2021_333_MOESM1_ESM.docx]

Additional file 1. Rate of misdiagnosis of severe psychiatric disorders at Amanuel Mental Specialized Hospital
